# Supplementary material for: Eco-Friendly Epoxy-Terminated Polyurethane-Modified Epoxy Resin with Efficient Enhancement in Toughness
Source: Polymers (Basel). 2023 Jun 24;15(13):2803. doi: 10.3390/polym15132803 (PMC10346895; doi:10.3390/polym15132803)
Supplement: Supplementary file 1 [file polymers-15-02803-s001.zip › polymers-2439099-supplementary.pdf]

Supporting information

Eco-Friendly Epoxy-Terminated Polyurethane-Modified Epoxy Resin with Efficient Enhancement in Toughness

Kun Zhang, Jinrui Huang *, Yigang Wang, Wenbin Li and Xiaoan Nie *

Key Laboratory of Biomass Energy and Material, Jiangsu Province, Co-Innovation Center of Efficient Processing and Utilization of Forest Resources, Key Laboratory of Chemical Engineering of Forest Products, National Forestry and Grassland Administration, National Engineering Research Center for Low-Carbon Processing and Utilization of Forest Biomass, Institute of Chemical Industry of Forest Products, Chinese Academy of Forestry, Nanjing 210042, China

* Correspondence: huangjinruiahu@126.com (J.H.); niexiaoan@icifp.cn (X.N.)

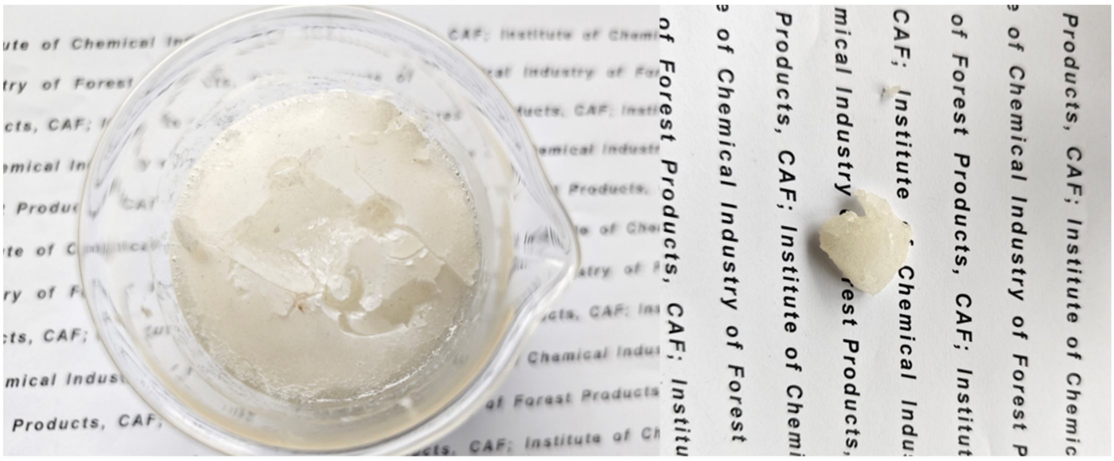

Figure S1. Photograph of epoxy with 50 wt % ITPU. Liquid epoxy resin quickly transferred into a solid after the addition of 50 wt % ITPU.

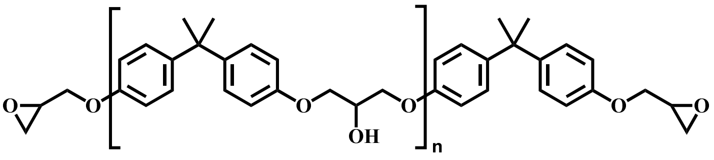

DGEBA

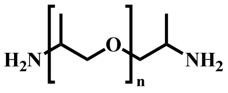

D230

Figure S2. Chemical structures of DGEBA and D230.

Table S1 Mechanical properties of cured epoxy samples

Sample	Elongation at break (%)	Toughness (MJ·m^{-3})	Tensile strength (MPa)	Young's modulus (MPa)	Flexural strength (MPa)
EP	6.58±0.23	3.29±0.24	62.75±1.16	3222±116	117.9±1.3
EPU10	11.02±0.37	4.22±0.36	50.11±1.00	2695±90	96.7±1.8
EPU20	17.22±0.55	4.81±0.40	34.06±1.14	2119±100	74.1±0.9
EPU30	30.16±0.77	5.31±0.41	19.32±0.73	1325±77	45.3±0.6
EPU50	55.42±1.36	5.71±0.48	13.16±0.49	403±48	17.0±0.5
ITPU10	5.54±0.42	3.05±0.20	69.31±1.55	3004±61	109.6±1.5
